# Host and Pathogen Communication in the Respiratory Tract: Mechanisms and Models of a Complex Signaling Microenvironment

**DOI:** 10.3389/fmed.2020.00537

**Published:** 2020-09-10

**Authors:** Samuel B. Berry, Amanda J. Haack, Ashleigh B. Theberge, Susanna Brighenti, Mattias Svensson

**Affiliations:** ^1^Department of Medicine, Center for Infectious Medicine, Karolinska Institutet, Karolinska University Hospital, Stockholm, Sweden; ^2^Department of Chemistry, University of Washington, Seattle, WA, United States

**Keywords:** pulmonary cross-talk, soluble factors, juxtacrine signaling, volatile signaling, *in vitro* models

## Abstract

Chronic lung diseases are a leading cause of morbidity and mortality across the globe, encompassing a diverse range of conditions from infections with pathogenic microorganisms to underlying genetic disorders. The respiratory tract represents an active interface with the external environment having the primary immune function of resisting pathogen intrusion and maintaining homeostasis in response to the myriad of stimuli encountered within its microenvironment. To perform these vital functions and prevent lung disorders, a chemical and biological cross-talk occurs in the complex milieu of the lung that mediates and regulates the numerous cellular processes contributing to lung health. In this review, we will focus on the role of cross-talk in chronic lung infections, and discuss how different cell types and signaling pathways contribute to the chronicity of infection(s) and prevent effective immune clearance of pathogens. In the lung microenvironment, pathogens have developed the capacity to evade mucosal immunity using different mechanisms or virulence factors, leading to colonization and infection of the host; such mechanisms include the release of soluble and volatile factors, as well as contact dependent (juxtracrine) interactions. We explore the diverse modes of communication between the host and pathogen in the lung tissue milieu in the context of chronic lung infections. Lastly, we review current methods and approaches used to model and study these host-pathogen interactions *in vitro*, and the role of these technological platforms in advancing our knowledge about chronic lung diseases.

## Introduction

Cell-mediated signaling events drive a wide range of physiological functions, ranging from tissue repair and homeostasis to immune response and disease, all of which occur in the complex biological environment of the pulmonary tract (1–3). Within the pulmonary tract, the exchange of cell-derived signals is implicated in the development, progression, and maintenance of infection (both acute and chronic), yet the nature of many signaling interactions remains unknown or unclear in both healthy and pathogenic environments (2–5). The severity and progression of lung infections are frequently examined within the scope of factors such as the pathogen-specific mechanism of infection, patient physiology (e.g., immune and vaccination status, age, transplants, etc.), and microenvironment composition (6–10), with the ultimate goal of understanding the role of various microenvironmental and mechanistic components on the pathogenesis of a singular infection. However, understanding the effects of these components, even within the context of a singular infection, is exceedingly difficult as there is immense variation in disease manifestation and outcome, contributed to in part by the complex signaling environment found in the lung. Chronic pulmonary infections (CPIs), broadly characterized by pathogen persistence and immune evasion as the pathogen adapts to or manipulates its microenvironmental niche, illustrate this difficulty, as CPIs can manifest in a myriad of forms resulting from microbial persistence after medical treatment, external exacerbation of underlying conditions (e.g., latent infection), shifts in internal microenvironmental cues, or potentially other understudied areas of the microenvironment, such as the lung microbiome (6, 10–14). The complexity of these pulmonary cell-signaling environments, coupled with the numerous external variables driving chronic infection, underscores the difficulty in accounting for the simultaneous effects of all of these different factors, including understanding how these host-host, host-pathogen, and pathogen-pathogen communications drive immune response and propagate infection.

The ability to interpret the chemical and biological communication in the lung microenvironment enables us to better comprehend the vital conversations that drive the disease state in chronic infections. However, the translation of these intrinsic signals to actionable immune defense can often confound the host biology, as pathogens have evolved numerous mechanisms to evade host defense systems and confound host communication. Further, this communication, which includes the release of signaling factors (e.g., soluble factor or volatile signaling) and physical interactions (e.g., juxtacrine signaling), acts cumulatively to simultaneously promote many different aspects of the infection ranging from epithelial adhesion and invasion by *Burkholderia pseudomallei* to secretion of *Aspergillus fumigatus* growth-promoting volatiles by *Pseudomonas aeruginosa* (15, 16). Understanding how signaling pathways in the lung microenvironment are shifted and manipulated in response to specific stimuli can elucidate key insight into the infection mechanisms of numerous pathogens, and provide information that can lead to novel diagnostics and treatments.

In this review, we focus on how chemical and biological signaling events in the lung microenvironment unfold during chronic lung infections, including recurrent, persistent, and latent infections with *Mycobacterium tuberculosis, Aspergillus fumigatus, Pseudomonas aeruginosa, Streptococcus pneumoniae, Staphylococcus aureus, Cryptococcus neoformans*, and *Burkholderia pseudomallei*. These host-pathogen interactions use analogous modes of cellular signaling and communication in the lung that involves soluble factor paracrine signaling (e.g., cytokines), juxtacrine signaling [e.g., pattern recognition receptors (PRR)], and volatile signaling [e.g., volatile organic compounds (VOC)], oftentimes with overlap of specific signaling mechanisms or components. We highlight similar signaling mechanisms across a spectrum of chronic pulmonary pathogens to create novel comparisons between diseases and to encourage interdisciplinary studies that translate methodologies, perspectives, and understanding for these infections. Further, we describe recent innovative *in vitro* technologies and platforms, in which the researcher has control over the complexity of the system, and suggest their use to examine the complex communication involved in the lung microenvironment during chronic lung infections.

## Infections and Cross-Talk at the Molecular Level

**Figure 1:**
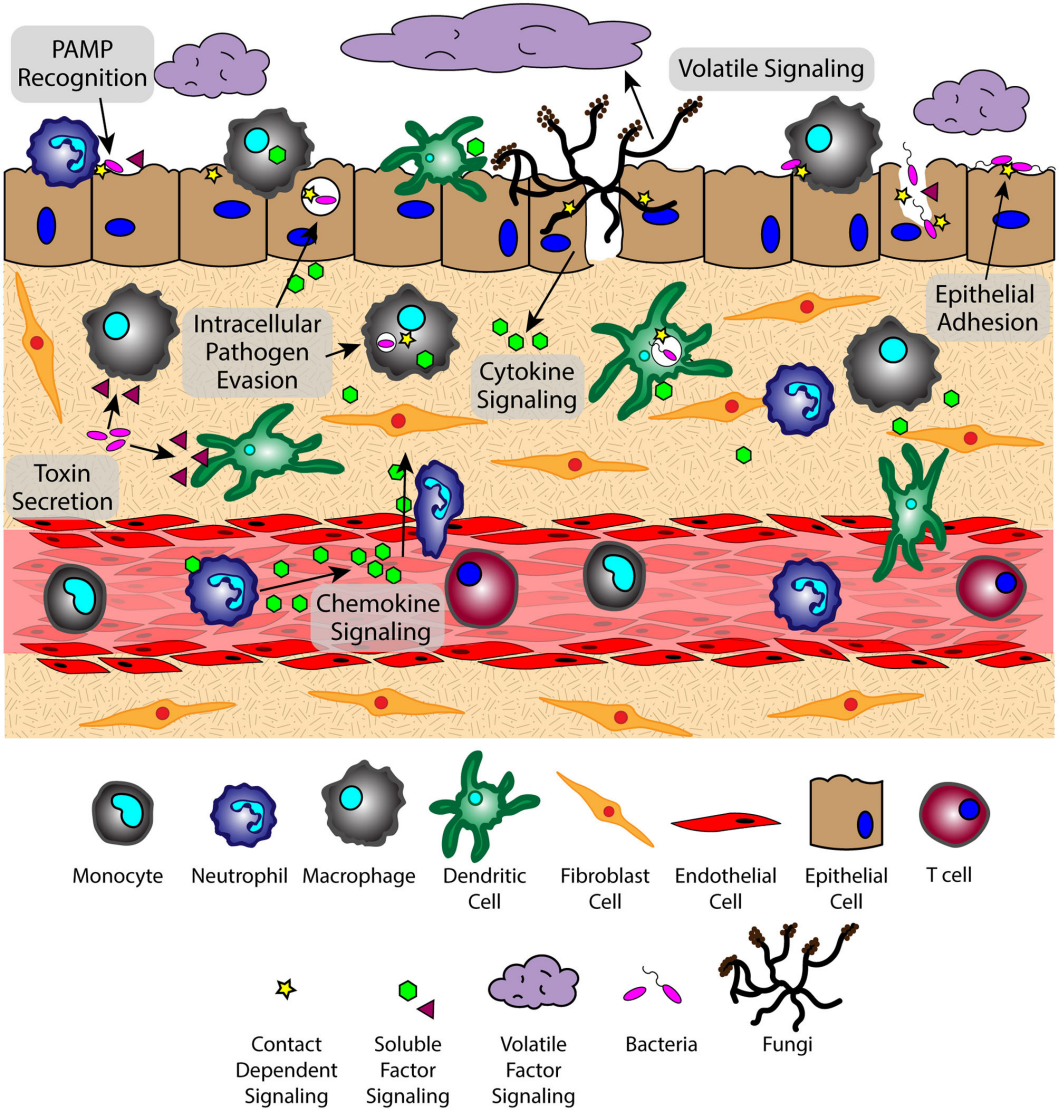
The lung microenvironment is home to a complex signaling network that interprets factors from various sources simultaneously. Signaling phenomena includes the exchange of soluble, volatile, and physical signals between cells of the host immune system, microbes, and host tissue components at varying spatiotemporal concentrations. Translating this complex quagmire of signals requires a broad perspective to see the role of each type of communication in the context of host-pathogen, pathogen-pathogen, and host-host signaling.

The lung microenvironment is a heterogenous interface consisting of innate immune cells (e.g., alveolar macrophages, neutrophils, monocytes, and dendritic cells), mucous-producing and ciliated epithelial cells, rare immune populations [e.g., mucosal-associated invariant T cells (MAIT cells) (17, 18), innate lymphoid cells (ILCs) (19–21)], commensal and pathogenic microbes, and underlying tissue and vascular networks that are all in constant reciprocal communication (Figure 1). This tissue organization, which supports multiple cell populations with vastly different environmental requirements, must parse through the signaling milieu of soluble, volatile, and physical cues to identify the biological and chemical stimuli needed for proper respiratory function and effective immune defense. However, upon pathogen challenge, the lung microenvironment faces new obstacles in converting these stimuli into a coherent immune response, as oftentimes pulmonary pathogens simultaneously use multiple modes of communication (e.g., physical, soluble) to evade immunity and propagate infection; for example, *S. pneumoniae*-derived neuraminidase (NanA) cleaves mucins on the epithelial surface to enable pathogen attachment on host glycoconjugates, while subsequently secreting pore-forming toxin pneumolysin to disrupt the epithelium (7, 22, 23). The inability of the host to respond to these pathogen challenges leads to an impaired microenvironment where slight deviations from signaling equilibria can promote a chronic condition (24, 25), highlighting the importance of understanding the signaling landscape in CPIs.

## Soluble Factor Paracrine Signaling

### Virulence Factors—Toxins

Pathogenic microbes employ a wide range of soluble virulence factor signaling mechanisms to combat the host immune response and persist *in vivo*, the most notable of which includes the use of host-targeting toxins. These toxins function in the early stages of infection to directly cause damage to the host microenvironment and create a favorable niche for the pathogen to survive. For example, *S. pneumoniae* makes use of a potent pore-forming toxin, pneumolysin, that binds to membrane-bound cholesterol and perforates the host cell, causing cell death through multiple mechanisms including destruction of DNA and cell cycle arrest (26). Pneumolysin has significant cytolytic effects and acts to induce the complement pathway and a proinflammatory cytokine response, leading to the degradation of barrier function in the host epithelium, pathogen uptake via platelet-activating factor receptor (PAFr)-mediated endocytosis, and pathogen trafficking across the epithelium into the underlying tissue for *S. pneumonia* dissemination (7, 27–29). Another prominent pathogen-derived toxin is the pore-forming compound α-hemolysin (α-toxin) utilized by *S. aureus* in pulmonary infection. A-toxin acts via binding to its specific cell receptor, ADAM10, wherein it oligomerizes to form the cytolytic pore and induce host cell lysis (30). The degradation of epithelial barrier integrity enables *S. aureus*, in a manner similar to *S. pneumoniae*, to invade into the underlying tissue, or to reside within the epithelial layer itself, where internalized bacteria can survive within a quasi-protective niche (31, 32). This intracellular residence permits the pathogen to enter into a dormant state protected from the host immune response and antibiotic therapy, contributing to its chronicity and enabling recurrent *S. aureus* infection (32).

The effect of *S. aureus*-derived α-toxin can be amplified during coinfection as well. Polymicrobial infections with fungal pathogen *C. albicans* activates the *agr* quorum sensing system in *S. aureus*, which leads to enhanced α-toxin secretion and decreased host survival, demonstrating how coinfection not only adds complexity to the lung microenvironment but also promotes increased virulence (33). Further, invasive fungal species, such as *A. fumigatus* and *C. neoformans*, are armed with a variety of toxins that impede or interfere with intracellular functions required to mount an effective immune response. *A. fumigatus*, one of the most successful opportunistic fungal pathogens, has a wide array of immunosuppressive secondary metabolites and toxins at its disposal including trypacidin, endocrocin, and gliotoxin (34–38). These toxins confer protection to *A. fumigatus* against numerous components of the host immune system. Trypacidin, found to localize to fungal conidia, induces high levels of nitric oxide (NO) and hydrogen peroxide (H_2_O_2_) production in bronchial epithelial cells, leading to severe oxidative stress and cell necrosis (34). Similarly, endocrocin, another spore-derived fungal toxin, inhibits neutrophil migration and displays significant virulence in a *Drosophila* model of infection (35). The most widely characterized *A. fumigatus* toxin, gliotoxin, has been found to inhibit multiple antimicrobial functions (e.g., superoxide defense) in a wide range of leukocytes, in addition to inducing apoptosis and inhibiting NFκB signaling, providing *A. fumigatus* with a broad repertoire to defend itself against a range of host immune responses (39–42); for a detailed review of gliotoxin's extensive immunomodulatory effects, refer to (43). The culmination of the effects of this library of toxins enables *A. fumigatus* to survive continual immune assault and create an environment favorable for colonization and proliferation.

### Virulence Factors—Immune Modulators

In addition to toxins, there are other virulence factors used to evade host immune responses and confound the cross-talk required for effective pathogen clearance; these factors are a series of distinct compounds ranging from surface-expressed cell wall components to quorum sensing (QS) molecules used for intermicrobial communication. During infection with *M. tuberculosis*, the pathogen utilizes alternative strategies to manipulate immune cells in the local tissue environment, such as expression of the type VII secretion system, ESX-1 (44). This secretion system transports potent early secreted antigen target 6 kDa (ESAT-6) and culture filtrate protein (CFP-10) that are essential for phagolysosomal escape into the cytosol and induction of host cell necrosis (44–47). This event will trigger a pro-inflammatory reaction and secretion of MMP9 by epithelial cells and *M. tuberculosis*-infected macrophages, leading to the recruitment of uninfected macrophages and other immune cells to contain the infection in a granuloma (48, 49), although the arrival of uninfected cells may be exploited by pathogenic mycobacteria to further disseminate the infection (50–52). The extent of virulence, and the capability of the mycobacteria to evade the host immune response, has been directly linked to the presence and function of ESAT-6 and other RD1/ESX-1 derived virulence factors, as avirulent strains lack this secretion system (53–55). The exact mechanism of action of ESAT-6 is still somewhat unclear, yet it has been recently found that the ESX-1 secretion system does not completely depend on ESAT-6 for membrane lysis and that its function is contact dependent, despite ESAT-6 being able to disrupt lysosome membranes at an acidic pH (45, 56).

Recently, QS molecules have been identified as another group of potential virulence factors capable of programming pathogen populations to evade immune responses, as well as to inhibit or manipulate the host immune response directly (57). This mechanism is widely observed in the case of *P. aeruginosa*, where quorum sensing molecule N-3-oxo-dodecanoyl-L-homoeserine lactone (3O-C_12_-HSL), which regulates the LasR QS circuit and numerous other virulence factors, acts as a virulence factor itself that inhibits the production of proinflammatory cytokines (e.g., TNF, IL-12) and the proliferation and differentiation of T cells (57, 58). Additionally, 3O-C_12_-HSL does not only target human cells, in which 3O-C_12_-HSL often interacts with the intracellular activation protein IQGAP1 (57), but also commensal organisms. In a mixed coculture with opportunistic fungal pathogen *C. albicans*, 3O-C_12_-HSL was found to inhibit filamentation of the fungi, indicating that *P. aeruginosa* can use its QS molecules to influence the host as well as other microbes in its surrounding microenvironment (59). This dual functionality of QS molecules is observed with other *P. aeruginosa*-derived factors, such as *Pseudomonas* quinolone signal (PQS), which dysregulates host immunity through manipulation of cytokine production and reduction of antibacterial activity of host immune cells (60, 61). The ability of a pulmonary pathogen to utilize QS molecules for more than one function (i.e., to self-regulate and evade immunity) supports their persistence against constant immune challenge while maintaining the ability to proliferate and survive. Other virulence factors take on a more traditional or singular role in establishing a pathway for infection. *C. neoformans*, a fungal species often associated with meningitis in immunocompromised patients, is capable of establishing a chronic lung infection in immunocompetent patients through the secretion of host-targeting enzymes (e.g., phospholipases, proteases) that degrade host immune components (e.g., phagocytic compartment membranes, lung surfactant) (62–66). These secreted virulence factors drive the ability of *C. neoformans* to survive and protect itself from initial immune responses until it adapts to the host environment and takes on phenotypic forms to support its persistence in a dormant state (64, 67, 68). The secretion or release of virulence factors, which can encompass different classes of molecules, mediates various anti-immune behaviors to create favorable niches for persistence; further, the presence of multifunctional molecules, such as QS molecules, adds to the complex milieu present in the lung microenvironment and demonstrates the importance of a broad perspective encompassing not only host-pathogen communication but also signaling amongst bacterial and/or fungal kingdoms.

### Cytokines

**Figure 2:**
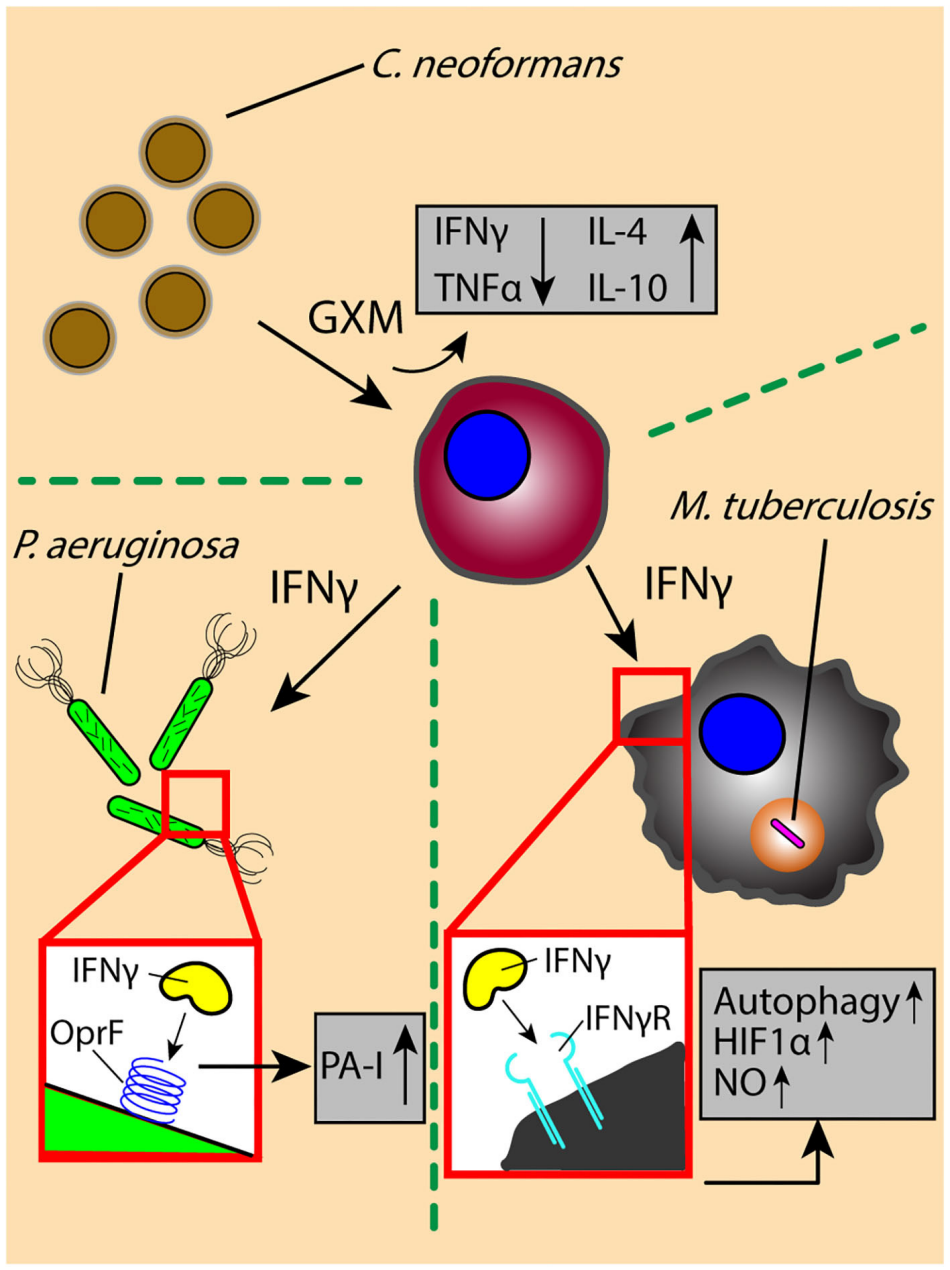
The exchange of soluble factors through paracrine signaling generates context-dependent effects. The secretion of proinflammatory factor IFNγ by T cells induces antimicrobial functions (e.g., autophagy) and signaling (e.g., HIF1α-mediated NO production) in *M. tuberculosis*-infected macrophages, while increasing the virulence of *P. aeruginosa* through OprF-binding and downstream induction of virulence factor PA-I. The secretion of IFNγ is decreased by interaction with *C. neoformans* virulence factor GXM, which decreases secretion of inflammatory factors (TNFα) while increasing secretion of anti-inflammatory factors IL-4 and IL-10. OprF, outer membrane porin F; PA-I, type I *P. aeruginosa* lectin; HIF1α, hypoxia inducible factor-1 α; NO, nitric oxide; GXM, glucuronoxylomannan.

Pathogen-derived soluble factors contribute partially to the cross-talk events in the lung microenvironment; host-derived signals are also present, often in response to or to defend from pathogen-derived factors. Cytokines are a principle form of secreted soluble factor used to trigger the immune system and induce diverse effector functions upon pathogen challenge; careful regulation of these cytokines is vital, as impaired or excessive cytokine signaling can lead to pathogen survival and persistence, or pathological tissue destruction in the lung, respectively. *M. tuberculosis*-infected macrophages secrete multiple inflammatory cytokines, including TNF, IL-1β, and IL-6, which induce an early proinflammatory response that promotes immune cell activation and migration and results in the generation of a granuloma. Activated T cells producing IFNγ initiate antimicrobial responses, such as the induction of NO and autophagy, to promote mycobacterial clearance (Figure 2) (69–74). However, disruption of IFNγ signaling due to a shortage of activated effector T cells or an increase in regulatory T cells can lead to pathogen persistence within infected macrophages and increased bacterial burden in the lung (75–78).

Contrary to the role of IFNγ in *M. tuberculosis* infection, there is evidence suggesting that the immune activating effects of IFNγ can lead to an increase in virulence by *P. aeruginosa*, wherein IFNγ binds to *P. aeruginosa* surface receptor OprF to induce downstream expression of virulence factor PA-I via its QS signaling network (79) (Figure 2). This unintended response to a host cytokine illustrates that cytokine signaling can have dual, context-dependent outcomes. In addition to intercepting host signaling molecules, pathogens can also manipulate the expression and secretion of host factors to alter the signaling environment in their favor. The *C. neoformans-*derived virulence factor glucuronoxylomannan (GXM) increases secretion of immunomodulatory factors IL-10 and IL-4 while downregulating pro-inflammatory compounds TNF and IFNγ in stimulated CD4+ T cells, driving a permissive immune response that supports increased yeast cell growth (Figure 2) (80); the manipulation of the secretion ratio of IL-4 and IFNγ induce a range of macrophage phenotypes—ranging from classically activated to alternatively activated and intermediate—that simultaneously contribute to antimicrobial and acquiescent outcomes (81). Similarly, macrophages infected with *M. tuberculosis* secrete increased levels of VEGF while granuloma-associated macrophages have an increased expression ANG-2; the increased levels of VEGF and ANG-2 correlate with increased vascularization and vessel permeability around the site of infection, respectively, which is subsequently used for pathogen dissemination and host manipulation (82, 83). Chronic pulmonary pathogens can not only usurp the balance of pro- and anti-inflammatory cytokines found in the signaling microenvironment to alter the type of immune response, but also influence the surrounding tissue infrastructure to its own benefit (84).

### Chemokines

The manipulation of host signaling by pulmonary pathogens also extends to chemokine signaling, which is a vital process for the recruitment of immune cells to the site of infection. Early in the course of pulmonary infections, the neutrophil-mediated pro-inflammatory response plays an important role in counteracting invading pathogens while also contributing to tissue pathology, making its manipulation an attractive target for pathogens. *S. aureus* utilizes multiple mechanisms to impede the recruitment of neutrophils to the site of infection by using secreted staphopain (i.e., cysteine proteases) that inactivates CXCR2 on neutrophils and blocks the attractant effects of infection-associated chemoattractants (85); additionally, *S. aureus* utilizes exoprotein SSL5 to scavenge chemokines and bind to chemokine surface receptors, effectively blocking the ability of neutrophils to respond to receptor-bound chemokines (86). Further, SSL5 is found to act on neutrophils through its capacity as an MMP-inhibitor, blocking potentiation of neutrophil chemokine IL-8 (CXCL8) and preventing neutrophil migration through collagen (87). The modulation of neutrophil recruitment is not limited to *S. aureus*-derived soluble factors, as *S. pneumoniae*-derived virulence factor PepO is found to contrastingly increase secretion of chemoattractants CXCL8 and IP-10 (CXCL10) from bronchial cell line BEAS-2B, suggesting that this factor may increase recruitment of neutrophils to the site of infection (88). T cell chemokine CXCL10 has also been associated with *Cryptococcus* infections; during murine infection with *C. gattii*, a close pathogenic relative to *C. neoformans*, an impaired T_H_1 migration in the lung is correlated with decreased *Ip10* expression and poor DC maturation when compared to *C. neoformans* infection, leading to an attenuated effector T cell response and increased pulmonary infection (89).

### Antimicrobial Molecules

Beyond host-host signaling (i.e., paracrine cytokine and chemokine signaling), the role of host-secreted factors directed toward pathogens is an essential functionality in clearing pathogens and preventing chronicity. Host cells utilize an array of armaments to directly kill pathogens, such as reactive oxygen and nitrogen species (ROS, RNS, respectively), cytotoxic molecules (e.g., granzymes, lysozymes, and perforins), antimicrobial peptides (e.g., human cathelicidin, LL-37, defensins, and granulysin), and antimicrobial small molecules [e.g., extracellular vesicles (EVs), miRNA]. ROS play a vital role in the clearance of pathogenic microbes, but must also be tightly controlled to minimize the risk of host-tissue damage via extensive oxidation, as well as host immune suppression at the site of infection. The tight regulation of this defense is evident in the ability of neutrophils to induce different types of ROS signaling based on the size of the microbe it encounters, triggering intracellular ROS against ingested small yeast cells while secreting extracellular ROS against the larger hyphal form of fungi (90). In contrast, pathogens can activate this defense in their favor through toxin secretion, such as *P. aeruginosa*-derived pyocyanin, which triggers neutrophil apoptosis through inducing ROS and disrupting mitochondrial membrane potential (91). However, ROS-mediated pathogen clearance is not limited to neutrophils, as plasmacytoid DCs are also found to utilize ROS to inhibit the growth of *C. neoformans in vitro*, although they remain less effective at fungal killing than conventional DCs (92).

In *B. pseudomallei* infection, bacteremic patients were found to have increased levels of pro-apoptotic compounds granzyme A and B (93); further, stimulating patient-derived monocyte-derived DCs with *B. pseudomallei* antigens LolC and Hsp60 led to downstream activation of CD8+ T cells and increased granzyme B secretion (94). The increased secretion of granzyme B likely plays a role in patient survival, as NK-cells from deceased patients are characterized by significantly lower levels of granzyme B expression (95). Similarly, control of intracellular *M. tuberculosis* has been shown to involve CD8+ cytotoxic T cells using the pore-forming protein perforin to enable the entrance of granulysin into infected cells and the subsequent killing of *Mycobacterium* through osmotic lysis (96). Accordingly, a reduced expression of perforin and granulysin in *M. tuberculosis* infected tissues have been shown to correlate with disease progression (97, 98).

More recently, work has focused on identifying the antimicrobial effects of other components found in the extracellular communication milieu, such as extracellular vesicles (EVs) or metabolic components. For example, in interactions between *A. fumigatus* and host immunity, both host and pathogen secrete bioactive EVs that target its respective counterpart and generate proinflammatory and antimicrobial responses (99, 100). Important components of these EVs, such as miRNA, are also involved in mediating intra- and inter-cellular immune mechanisms and responses (101, 102), and are implicated as potential biomarkers of infections such as tuberculosis (103). Further, the metabolic cross-talk between pathogens, commensal organisms, and hosts is believed to be an influential factor in a number of relevant infection models. Indeed, many pulmonary pathogens such as *A. fumigatus* and *P. aeruginosa* are known to secrete siderophores that chelate iron from the host for their own use (57, 104, 105). Additionally, nitrogen and sulfur availability is thought to significantly contribute to pathogen survival, where availability of nutrients such as tryptophan and asparagine can modulate pathogen behavior and metabolism, as well as influence host immunity. We refer readers to these excellent recent reviews on the role of metabolic cross-talk in infections (106, 107).

## Physical Contact Juxtacrine Signaling

Contact dependent signaling, or juxtacrine signaling, complements soluble factor signaling and is a crucial component of the intercellular signaling microenvironment. Contact-dependent signaling is one of the first modes of communication used to detect pathogens in the lung. Innate immune cells, such as macrophages and dendritic cells, rely on the expression of germline-encoded receptors [e.g., pathogen recognition receptor (PRR)] to sample and detect pathogen-specific molecules [e.g., pathogen associated molecular patterns (PAMPs)]. For example, surface bound receptors toll-like receptor (TLR) and c-type lectin receptor (CLR) detect conserved pathogenic molecules such as lipopolysaccharide (LPS) on the surface of gram-negative bacteria *P. aeruginosa* and *B. pseudomallei*, and β-glucans on fungi such as *A. fumigatus*, respectively (108–110).

### Pathogen Recognition Receptors and Associated Molecular Patterns

#### Toll-Like Receptors (TLRs)

The TLR-signaling axis is present in a wide range of infections, including tuberculosis, where innate immune cells expressing TLR2 are able to detect multiple *M. tuberculosis* surface components (111), such as LpqH (112), LprG (113), and LprA (114), which impede intracellular signaling pathways and interfere with MHC II antigen processing and presentation. TLR4 detection of intracellular *M. tuberculosis* modulates intracellular signaling pathways governing the immune response in an antigen-dependent manner. Specifically, TLR2 detection of cell wall components PIM and mannosylated lipoarabinomannan (Man-LAM) result in robust anti-inflammatory responses characterized by decreased secretion of TNF and IL-12p40 and increased secretion of IL-37, respectively (115, 116). At the same time, HSP70-cofactor GrpE activates DCs and leads to their subsequent preferential induction of proinflammatory Th1 cells via TLR4 recognition (117). Additionally, detection of Man-LAM by different macrophage receptors such as the mannose receptor, complement receptors, and DC-SIGN can manipulate induction of proinflammatory responses to reduce local immune cell activation, as well as to inhibit phagosomal maturation inside infected macrophages (118–120).

#### C-Type Lectin Receptors (CLRs)

In conjunction with TLR pathogen sampling, CLR detection mechanisms also complement the innate immune system's ability to detect a wide range of pathogens. CLRs Dectin-1 and DC-SIGN have been implicated in the recognition, binding, and phagocytosis of *A. fumigatus* (121, 122), as single nucleotide polymorphisms in genes coding for Dectin-1 and DC-SIGN were associated with a higher risk of invasive pulmonary aspergillosis (123). Further, the absence of Dectin-1 ligand β-1,3-glucan on the surface of *A. fumigatus* leads to a more efficient complement-mediated antifungal immune response driven by DCs; this is found to be due to improved complement binding in the absence of β-1,3-glucan, and that galactomannan binding via DC-SIGN drives a proinflammatory cytokine response (124). However, *A. fumigatus* leverages other cell wall components to drive virulence, such as DHN melanin, which is able to block LC3-associated phagocytosis and subsequent fungal killing through impeding of NADPH oxidase from entering the phagosome (125). Host immunity is able to combat the virulence factor DHN melanin through binding via the melanin-sensing C-type lectin receptor (MelLec) found on endothelial cells and myeloid cells, the mutation of which is found to increase the susceptibility of stem-cell transplant patients to aspergillosis and to decrease proinflammatory cytokine secretion in macrophages (126).

The interactions between these PRRs and PAMPs strongly activate immune cells and initiate intra- and intercellular signaling to recruit other immune cells and clear the pathogen; however, this interaction is also vulnerable to manipulation by pathogens through suppression of PRRs or masking of PAMPs to avoid detection (104). Similar to *A. fumigatus, C. neoformans* utilizes L-DOPA melanin as a defense against oxidative stress from phagocytic immune cells (127–129), yet it relies mostly on its complex capsule to evade detection by host PRRs. *C. neoformans* contains a heterogenous multi-layered polysaccharide capsule that can block immune detection of fungal PAMPs, such as β-1,3-glucan, while preventing the adherence of complement proteins or antibodies to the surface (130). To further protect itself, *C. neoformans* can alter the physical characteristics of the capsule to adapt to environmental cues (131, 132), and capsule component glucuronoxylomannan (GXM) can induce L-selectin shedding and decreased TNFR expression on neutrophils, likely impeding their ability to bind to cell surfaces at the site of infection (133).

### Epithelial-Pathogen Interactions

#### Pathogen Adhesion to Epithelium

Pulmonary epithelial cells play multiple roles in maintaining lung immunity, ranging from creating and maintaining a physical mucous barrier to expressing TLRs and initiating immune responses via proinflammatory cytokine secretion (134–138). For a pathogen, epithelial cells often represent the first challenge that must be overcome in order to successfully establish an infection, thereby requiring passage through or destruction of epithelial cells (7, 31, 32). After entrance into the airway, pathogens must surmount the outer layer of the epithelium—containing mucous, surfactant, and scavenging immune cells—as this region is a hostile environment for the establishment of colonies or infections. Therefore, microbes have developed an array of physical methods to pass through the mucous layers and bind to the surface epithelium to prevent their clearance. The pathogen *B. pseudomallei* expresses genes *boaA* and *boaB*, which code for proteins comparable to the *Y. enterocolitis* adhesin YadA; these adhesin proteins have been found to significantly increase the adhesion of *E. coli* expressing *boaA* and *boaB* to alveolar and bronchial cell lines, and have been implicated the in intracellular survival of the pathogen (139). Further, this adhesive capability of *B. pseudomallei* can be complemented by the adaptation of a biofilm phenotype, which was found to promote greater adhesion and subsequent invasion and infection by the bacteria (140). Indeed, the ability of *B. pseudomallei* to adhere to the airway epithelium is directly correlated with its virulence and ability to induce cellular damage (141). The strength of the adhesion of *B. pseudomallei* to the airway epithelium is also correlated with the expression of genes regulating QS and virulence pathways (142, 143); these correlations underscore the importance of pathogen adhesion for initiating a successful infection. Similarly, *C. neoformans* utilizes secreted phospholipase B to improve its ability to adhere to pulmonary epithelial cells, likely through interactions between surfactant protein D (SP-D) and polysaccharides on the surface of the *C. neoformans* capsule (63); additionally, capsule components GXM and MP84 are known to facilitate the binding of different *C. neoformans* strains to airway epithelial cells (144), enabling *C. neoformans* to initiate infection at this primary immune barrier (145). SP-D binding is also found to play a role in the immune response against *A. fumigatus*, wherein SP-D binds specifically to melanin on the fungal conidia and associates with galactomannan and galactosaminogalactan on the fungal cell wall; further, SP-D bound conidia were phagocytosed more readily than un-opsonized conidia, and induced a greater transcription of proinflammatory cytokines (146).

#### Epithelial Defense Against Pathogens

To counter these various pathogen-driven mechanisms of infection, the host epithelium employs an expanded array of defenses that support its active role as an innate immune component in the airway microenvironment (29, 135–138, 147). These defenses includes the direct secretion of antimicrobial peptide (AMP) classes such as defensins (148–150), cathelicidins (150, 151), lysozymes (152, 153), lactoferrins (152–154), and secretory leukocyte proteinase inhibitor (155, 156), to induce immunity and assist in antimicrobial defense. Some of these AMPs are constitutively secreted, such human β-defensin-1, yet other β-family defensins and AMPs are secreted in response to juxtacrine signaling events, such as TLR activation or NF-κB signaling (135, 136, 148). While the lung epithelium can deploy some defense mechanisms against adherent pulmonary pathogens, once adhered, these pulmonary pathogens can invade into or through the epithelium to establish a protective niche for growth and persistence. Discovery of mechanisms or treatments to decrease this adherence and enable clearance from the lung epithelium prior to pathogen entry can have a significant impact on disease outcome.

#### Intracellular Cross-Talk

For many pathogens, passage through the epithelial barrier is unknowingly assisted by phagocytic immune cells. Physical detection of pathogens by PRRs can initiate an endocytic process to encapsulate the pathogen within an intracellular vacuole that then uses numerous mechanisms to clear its contents, such as vacuole acidification or fusion with vacuoles containing antimicrobial peptides (e.g., human cathelicidin, LL-37), antimicrobial molecules (e.g., RNS, ROS), or lytic proteins (e.g., cathepsins, phospholipases) (157). During *M. tuberculosis* infection, activated macrophages release a collection of reactive nitrogen and oxygen species generated via NOX2 into the phagosome to induce oxidative stress and destruction of microbial membranes and intracellular components (157). Induction of autophagy and xenophagy pathways in *M. tuberculosis*-infected macrophages provides an alternative mechanism of defense against the intracellular pathogen, and modulation of these pathways has been the target of host-directed-therapies (158). This intracellular communication between the host cell and pathogen enables a strong form of self-defense for the cell, as well as a method to clear an acute infection and potentially avoid chronicity. Yet, for many pathogens, including *M. tuberculosis*, the ability to escape the phagocytic vacuole is the key to survival and persistence and an important mechanism for immune evasion (159–162).

Though not commonly perceived as an intracellular pathogen, *S. aureus* utilizes several methods to persist within a protective intracellular niche, ranging from secreting factors to block the acidification of the phagosome in leukocytes to disrupting phagosomal membranes for escape into the cytosol in epithelial cells (163, 164). Indeed, *S. aureus* utilizes its presence inside PMNs to usurp PMN expression of “danger” signals and to manipulate the phenotype of innate immune cells when in coculture, ultimately altering the ability of macrophages to clear *S. aureus*—infected PMNs and enabling intracellular persistence of the bacteria (165, 166). *S. aureus* can evade and manipulate PMN cell (i.e., neutrophil) function and antimicrobial effects in different ways, which is reviewed in depth here (165). *S. aureus* also utilizes the intracellular space of nonprofessional phagocytes, such as epithelial cells, as a protective niche to hide from host immune cells and to diverge into a heterogeneous population of replicating invasive or non-replicating persistent phenotypes depending on its intracellular environment, host cell metabolism, and bacterial colony variance (32, 167).

**Figure 3:**
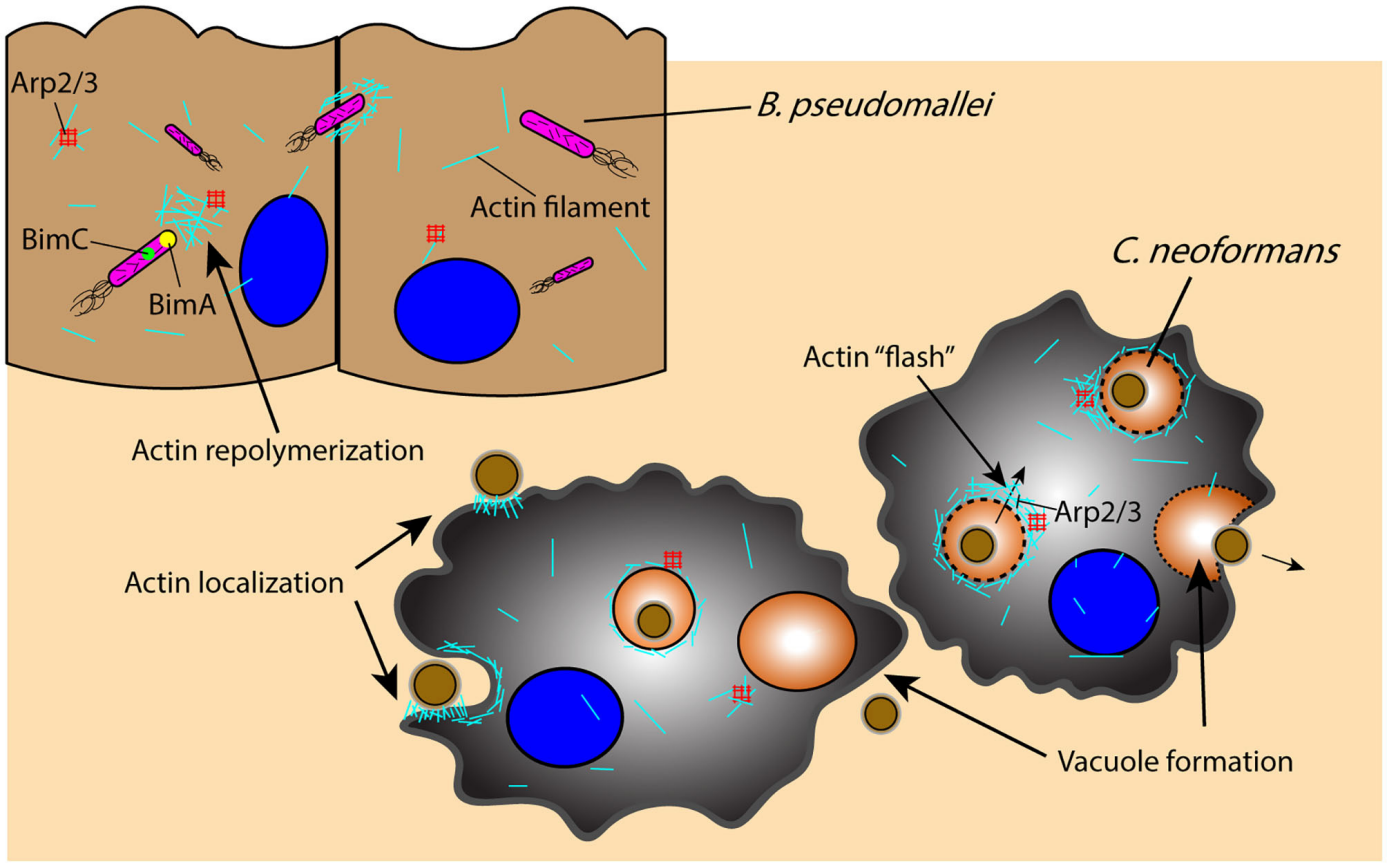
Pathogens leverage host cytoskeletal components for movement within and between host cells in response to host-initiated physical interactions. Actin-mediated movement of *B. pseudomallei* is vital for pathogen survival and movement subsequent to phagosomal escape, as proteins BimA and BimC enable actin repolymerization in an Arp2/3-independent manner and the ability to protrude and infect proximal cells. Similarly, actin localization to the phagosome is an important component of *C. neoformans* phagocytosis and containment, as actin undergoes an Arp2/3-dependent rapid polymerization (“flash”) in response to *C. neoformans* permeabilization of the phagosome; disruption of the phagosome and *C. neoformans* extrusion into neighboring cells or extracellularly is an actin-dependent process that results in vacuole formation. Arp2/3, actin-related proteins 2/3; BimA, *Burkholderia* intracellular motility A; BimC, *Burkholderia* intracellular motility C.

Once an intracellular pathogen manages to escape from the phagosome, it must rely on additional mechanisms for movement through the cell itself. An important component of this intracellular mobility is actin-based motility, which is utilized by a wide range of pathogens including *B. pseudomallei* and *C. neoformans* to manipulate the host cell cytoskeleton for their own movement and eventual dissemination through cell–cell spreading (Figure 3) (168, 169). Intracellular *B. pseudomallei* that have escaped the phagosome utilize bacterial proteins BimA and BimC to polymerize and remodel actin at its poles, enabling the pathogen to move through the cell to the outer membrane, from which it can protrude out of the cell and infect proximal cells (Figure 3) (168–170). *C*. *neoformans* similarly uses cell to cell spreading to infect neighboring cells while evading immune surveillance mechanisms, relying on an actin-dependent method for phagosomal escape and extrusion; however, the host cell utilizes actin flashes, or rapid repolymerizations, to temporarily prevent *C. neoformans* escape from the phagosome (Figure 3) (171, 172). The importance of actin in the pathogenesis of *C. neoformans* is also demonstrated by the dependence of macrophages on actin for the uptake and internalization of the extracellular yeast cells, which is significantly decreased in the presence of actin depolymerizing agents (173).

## Volatile Signaling

Whereas there exists a substantial amount of information regarding soluble factor signaling and juxtacrine signaling mechanisms, much less is known regarding volatile signaling. Principally regarded as a potential diagnostic for identifying bacterial species *in vivo* (174–176), volatile chemicals secreted by microbial species perform multiple functions as both symbiotic and antagonistic factors in complex signaling environments (177–180); indeed, microbial volatile organic compounds (mVOCs) have been described to play an important role in both intra- and inter-kingdom interactions (180). Additionally, these factors can originate from various microbes and kingdoms, and vary based on the microbial makeup of colonies, as well as the morphological form of microbe (i.e., spore vs. filamentous). Secreted mVOCs have been correlated with pathogen identity and even disease severity (181, 182), yet how these mVOCs influence pathogen behavior and host response is still widely unknown. We review mVOCs that target other commensal microbes and mVOCs that target host cells, as well as any downstream effects that result from this volatile cross-talk. However, volatile signaling between pathogens and the host lung is a vastly understudied area that represents a potentially significant wealth of information regarding host-pathogen interactions and as such requires further studies.

### Pathogen-Targeted Volatile Signaling

**Figure 4:**
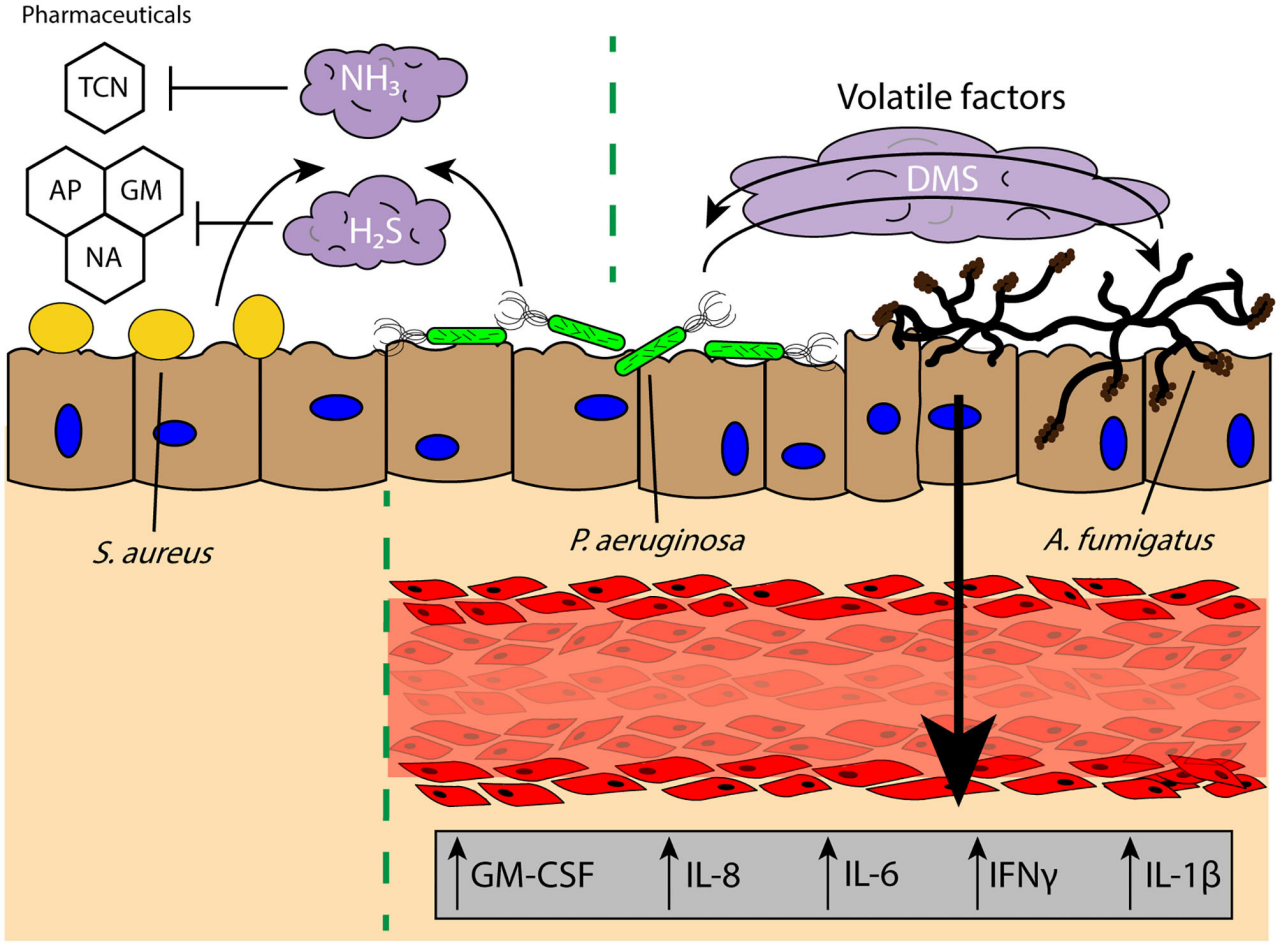
Volatile signaling in the lung microenvironment supports commensal and pathogenic microbial populations against diverse challenges and stimuli. Pulmonary pathogens *S. aureus* and *P. aeruginosa* secrete biogenic ammonia (NH_3_) and hydrogen sulfide (H_2_S) which offers protection against pharmacological interventions such as TCN and reactive oxygen species induced by GM, AP, or NA, respectively, to persist in the lung microenvironment. Further, the secretion of DMS from *P. aeruginosa* has been found to promote *A. fumigatus* growth, and the bidirectional exchange of volatile factors between the two pathogens leads to increased secretion of proinflammatory factors GM-CSF, IL-8, IL-6, IFNγ, and IL-1β from a lung tissue model of infection. TCN, tetracycline; GM, gentamicin; AP, ampicillin; NA, nalidixic acid; DMS, dimethyl sulfide.

Commensal microbes secrete volatile factors to promote synergetic interactions within an environment, wherein one microbe may secrete a mVOC that is then sequestered and metabolized by another or promote the growth of a neighboring microbe (177, 180). In the widely studied coculture system of *P. aeruginosa* and *A. fumigatus*, it has been found that volatile signaling between these two organisms results in stimulation of *A. fumigatus* growth from *P. aeruginosa*-derived dimethyl sulfide (Figure 4) (15); this result is contrary to what is observed when these two pathogens are in physical or shared media contact, where *P. aeruginosa* secretes toxins that inhibit the growth of *A. fumigatus* (183, 184). These interactions illustrate that the chemical communication between two organisms cannot be solely examined in one context and that growth conditions and the spatial distribution of microbes can yield different pathogen outcomes. This is further evident from the impact of oxygen availability on the *A. fumigatus* “volatilome,” which generates discernable volatile metabolite profiles dependent on the hypoxic state in the surrounding environment (185). The secretion of specific volatiles can also promote the survival and antibiotic resistance of pathogens, often to the benefit of the rest of the infectious colony. H_2_S is released by a wide range of pulmonary pathogens, such as *Bacillus anthracis, P. aeruginosa*, and *S. aureus*, and inhibition of H_2_S was found to increase the susceptibility of the bacteria to antibiotics (Figure 4) (186). The cytoprotective role of H_2_S is believed to result from its ability to control oxidative stress around the bacteria and prevent oxidation and DNA damage (186); this protection not only helps the bacteria resist antibiotic treatment, but also host-based oxidative stress (i.e., ROS). Antibiotic resistance can also be modulated by biogenic ammonia, which has been shown to improve resistance to tetracycline in *S. aureus* and *P. aeruginosa*, illustrating the impact of the volatile signaling landscape in the ability of a lung pathogen to survive and persist in a chronic infection (Figure 4) (187). This beneficial effect on growth and resistance is important for infections where there are multiple distal sites of pathogen colonies, as communication between these locations is not limited by the soluble diffusion- or contact-based signaling required for paracrine and juxtacrine signaling, making it more difficult for the host to intercept or impede these signals. However, much is still unknown regarding the detrimental and antagonistic effects of pathogen-derived volatiles on other pathogens in the lungs, as much of the research has focused on either the positive effects of microbial volatile signaling for pathogen survival, or the use of these volatile signals as diagnostics for bacterial colonization and infection (174, 175, 187).

### Host-Targeted Volatile Signaling

The effects of volatile communication between microbes and their host counterparts is even less studied due to difficulties associated with subjecting host cells to volatile chemicals in a biomimetic manner. Complex *in vitro* models can partially solve this difficulty, as cells are not cultured in a traditional submerged manner (188, 189). We discuss some of these *in* vitro models in subsequent sections. Pathogen-derived volatile signals received by host cells can vary extensively from QS molecules to mVOCs; these factors can then in turn manipulate the behavior of host barriers to infection. One well-characterized volatile compound that performs a range of pathogen functions is 2-amino acetophenone (2-AA), a QS-regulated compound secreted by *P. aeruginosa* and *B. thailandensis* (a close nonpathogenic relative of *B. pseudomallei*) that has been linked to a persistent chronic phenotype in *P. aeruginosa* (190). Pretreatment with 2-AA in *ex vivo* murine macrophages led to a decrease in proinflammatory cytokine secretion while modulating innate immune signaling pathways, which can decrease tissue pathology but lead to insufficient clearance of *P. aeruginosa in vivo* (191). Additionally, 2-AA can disrupt oxidative homeostasis and induce ROS-mediated oxidative stress and apoptosis signaling, which is believed to further aid in the establishment of an environment conducive to chronic infection (192, 193). While the diffusion of these factors into the host tissue has detrimental effects, the influence of pathogen-derived volatiles on the host epithelium and immune cells is much lesser-known. Some cytotoxicity studies have been conducted demonstrating the harmful effects of volatiles such as 1-decanol and 1-octen-3-ol. However, these studies used submerged cell cultures exposed to media containing the mVOC and lacking an air-exposure component, or subjected bacterial cells to the compounds for *umu* and Ames testing (194, 195). Overall, there is a substantial and immediate need for research in this field that relates the effect of pulmonary pathogen-derived volatiles to human host immunology and disease outcomes, as much of the fundamental characterization of this space is still missing (196).

## Modeling Pulmonary Infections and Cross-Talk

The role of cross-talk in pulmonary infections is beginning to be more understood as new analytical methods enable the examination of the cellular secretome (197–199) and novel *in vitro* lung tissue models facilitate scrutiny of the role of complex microenvironment conditions on lung pathology (178, 189, 200–202). Recapitulating lung tissue *in vitro* is challenging due to the complexity of the organ, which contains various components such as an air-exposed epithelium, a structurally intrinsic extracellular matrix, a complex vasculature network, and a myriad of immune components (203) (Figure 1), as well as a unique microbiome that contributes to the exchange of chemical signals in this microenvironment (6, 204, 205). The airway itself varies in shape and size, transitioning from the bronchi to bronchioles to the alveoli, reflecting a change in function from a conducting section to a gas exchange section (200, 202, 206), as well as regional differences in cell phenotypes and histology. For example, the small airways are lined with pseudostratified columnar ciliated epithelium with interspersed mucin secreting goblet cells; these features are functionally equipped to generate and move mucus, an important immune defense against extracellular microbes (136, 207, 208). In contrast, the alveoli contain a simple squamous epithelium, which is functionally equipped for the diffusion of gases between the alveoli and the surrounding capillary network (206, 209).

A significant challenge in the development of *in vitro* lung models is finding a balance between a simple and robust system suited for high throughput examination and a system that more closely recapitulates the full complexity of the human lung microenvironment. Perhaps the most significant power of *in vitro* systems is that they can be engineered along this spectrum, tailored to a particular level of complexity to answer a specific biological question. This configurability of an *in vitro* system, including choice of human cell types (e.g., epithelial, endothelial, stromal, immune cells), cell source (e.g., immortalized human cells or primary human cells), ECM components (e.g., collagen, matrigel, laminin), and two- or three-dimensional placement of these components relative to each other, grants the researcher unprecedented control over the microenvironment and the ability to manipulate the *in vitro* system (200, 210, 211).

Studies utilizing animal models have helped to develop much of our understanding of lung pathology and mechanistic biology. However, there are well-known limitations to animal models in fully elucidating the complex microenvironment and cross-talk between pathogens and the human tissue microenvironment, such as physiological immune system differences and differences in disease susceptibility and development. For example, mice do not become infected with *M. tuberculosis* in the same way as humans, as they form structurally different granulomas and do not commonly develop necrotizing lesions (212). However, other animal models do offer closer homology to humans for the study of CPIs, such as non-human primates or humanized mice (213–218), although these are more expensive, logistically demanding to use, and raise ethical concerns.

Innovative organotypic and tissue engineered models have incorporated key biological functions (219, 220), extracellular matrix (ECM) and structural components (188, 221, 222), mechanical stimuli (188, 221–225), and placement of pathogens with respect to lung tissue components to mimic *in vivo* interactions. These *in vitro* models allow researchers to hone in on specific aspects of the lung physiology and manipulate conditions to better understand the underlying signaling mechanisms governing the pathology of infection. Here, we describe the current landscape of *in vitro* lung models, with a focus on utilizing these models as a tool for studying the microenvironmental cross-talk in CPIs (Table 1). Many of these models have already elucidated key insights into CPIs, such as the dependence of early *M. tuberculosis* granuloma formation on ESAT-6 and the disruption of this granuloma formation with inhibition of tissue matrix metalloproteinases (MMPs) (49, 227). However, many models that were not explicitly designed for modeling infection have the potential to be adapted for studying cross-talk in infection. Further, many of these models can be kept in culture for extended periods of time (±4–8 weeks) (220–222, 226, 228), which may enable some investigation into long term host-pathogen interactions similar to that found in chronic infections. While we do not discuss *in vitro* organoid models here, we refer readers to these sources for lung organoid models (229–232).

**Table 1:** Current *in vitro* microfluidic platforms enable researchers to precisely control the microenvironment through incorporation of various cell types, ECM components, and mechanical stimuli, while maintaining compatibility with a wide range of established readouts.

***In vitro* Model Type**	**Advantages/Disadvantages**	**Specific examples**	**Applications for investigating cross-talk in infections**
Transwell/cell culture inserts	+ Simple and robust + Compatible with standard cell culture equipment + Standard air-liquid interface protocol + No external equipment requirement (e.g., pumps or vacuum for controlled fluid or air flow) + Compatible with established readouts (e.g., microscopy, RNA analysis) + Access to cultures for direct manipulation − Limited customization − Mechanically static − Poorly quantified diffusion gradients	(189) 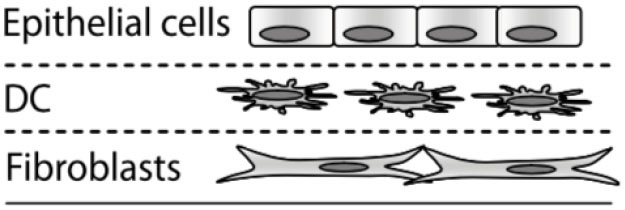	• Direct contact signaling • Soluble factor signaling • Co-culture with various cell types (e.g., DCs, macrophages, stromal cells) • Air-liquid interface on 3D culture • Cell-permeable inserts can be used for neutrophil/immune cell migration modeling through epithelium
Mechanically “breathing” microfluidic model	+ Cyclic airflow and mechanical stretching possible + Continuous nutrient/media flow + Customizable + ALI compatible + Compatible with established readouts (e.g., microscopy, RNA analysis) + Control over diffusion gradients − External equipment required (e.g., syringe pump for fluid flow control and vacuum pump for pressure control) − Microfabrication technology may be necessary for additional customization − Difficult to access cultures for direct manipulation	(188, 221)“Lung-on-a-Chip” 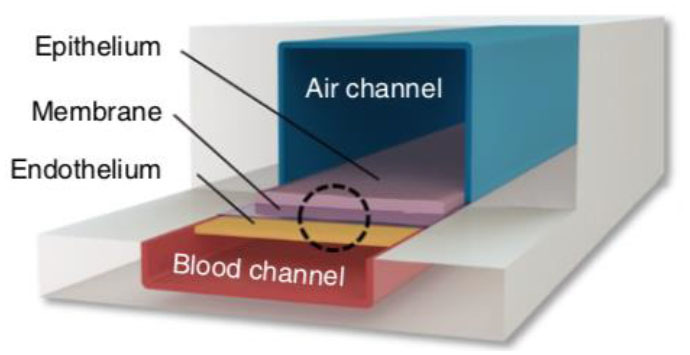	• Direct contact signaling • Soluble factor signaling • Investigations involving mechanical stretch • Investigations requiring controlled air or liquid flow • Coculture with multiple cell types (e.g., endothelium) • Porous membrane allows for neutrophil/immune cell migration modeling through epithelium • Control over airflow in ALI model
		(223) 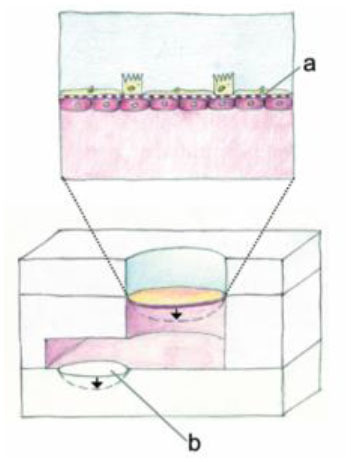	• Direct contact signaling • Soluble factor signaling • Investigations involving mechanical stretch • Investigations requiring controlled air or liquid flow • Coculture with multiple cell types (e.g., endothelium) • Porous membrane allows for neutrophil/immune cell migration modeling through epithelium • Open well for easier access of culture for downstream analysis
Hydrogel-based microfabricated models	+ Customizable + Complexity in tissue components and shape + Multiple signaling modes (i.e., volatile, direct contact, soluble factor, etc.) + ALI compatible + Open systems enable access to cultures for direct manipulation − External equipment may be required if controlled fluid flow or airflow is desired − Microfabrication technology may be necessary for additional customization	(178) 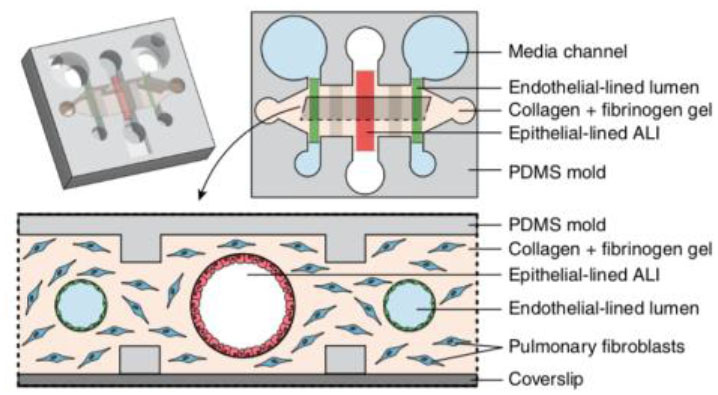	• Volatile signaling coculture experiments • Direct contact signaling • Soluble factor signaling • Investigations involving endothelial, mesenchyme, and epithelial cells • Modular cell culture experiments • Co-infection experiments
		(226) 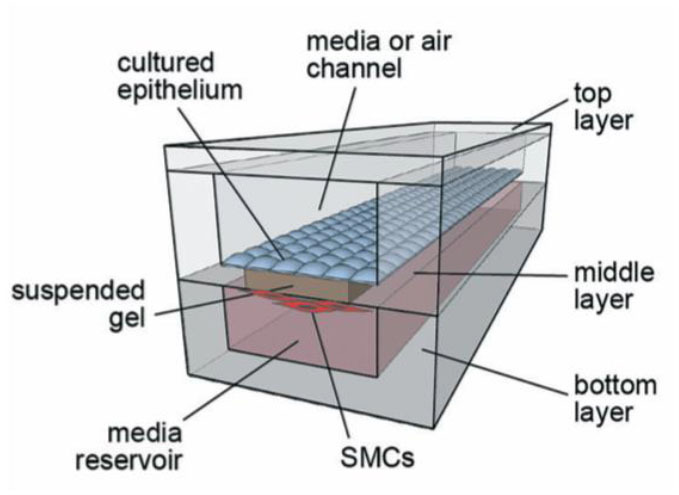	• Co-culture with epithelial and smooth muscle cells • Native ECM interactions (i.e., collagen/matrigel) • Continuous media flow • Air liquid interface • Soluble factor signaling • Direct contact signaling

*Various model types can be adapted for different biological systems, dependent on which components of the lung microenvironment need to be examined*.

## Transwell Inserts: A Simple, Robust Coculture Model

Advances in culturing techniques have led to an expansion beyond a submerged simple monolayer culture of lung epithelial cells, which fails to replicate tissue-level functionality (233–235). There is much interest and limited success in developing culturing conditions for inducing differentiation of easy to obtain cell lines into various other cellular phenotypes that are found in the airway (219, 220). For example, the establishment of an air liquid interface (ALI), which mimics the native environment of the lung epithelium, can differentiate a small-airway epithelial cell from a cobblestone monolayer culture into a mucociliary phenotype complete with ciliated pseudostratified columnar epithelium (219, 220) and mucin-producing goblet cells (220, 226, 236). Using other epithelial cell lines, an ALI can help create alveolar epithelial models, wherein these cell types can recapitulate alveolar function as measured by the increased surfactant secretion from type II-like alveolar epithelia (188, 237).

Widespread use of cell culture inserts technologies, such as Transwell inserts, have allowed for multiple monoculture and coculture experiments utilizing an ALI to differentiate epithelial cells into a biomimetic small airway model (219, 220, 238). The Transwell insert, produced by Corning Life Sciences, utilizes a simple and robust design that integrates into standard laboratory equipment. The insert consists of a permeable membrane that sits above the bottom of a standard tissue culture well. The membrane pore size can be chosen from 0.4 to 8.0 μm, with the larger pore sizes often used for cell migration assays, and the smaller pore sizes (typically 0.4 μm) used for coculture, soluble factor signaling, and creating ALI cultures. The inserts can also be used to establish an ALI in lung epithelial models (219, 220, 238) by culturing epithelial cells on the permeable membrane and removing the appropriate amount of media such that the cells are exposed to air on the apical (top) side, while still receiving nutrients through the permeable membrane on the basolateral (bottom) side. For coculture, cells can be cultured on top of the permeable membrane, and a different cell type can be cultured in the well-plate floor or even on the underside of the membrane, allowing the two cell types to communicate via soluble factor signaling through the porous membrane (239, 240). This functionality is particularly useful for studying cross-talk in infections.

### Transwell Models for Studying Pathogenic Infection

Due to the ease of use and efficacy of the Transwell cell inserts for establishing an ALI and a mucocilliary differentiated population, Transwell insert models have been utilized in several studies specifically for studying signaling involved in lung infections. The simplest model utilizes a single cell type (e.g., epithelial cells) cultured on the Transwell insert membrane to establish an ALI and a differentiated airway epithelium. The cells are then exposed to a pathogen of interest either by incorporating pathogen conditioned media into the bottom chamber (238) or by introducing the pathogen itself on the apical side of the airway epithelium (241, 242). An example of both these approaches can be seen in a study done by Halldorsson et al. (238) in 2010. This study took advantage of the ability to induce tight junction formation using an ALI produced by a Transwell insert to study the effect of azithromycin treatment for maintaining tight junction integrity during a challenge with live *P. aeruginosa* and bacterial culture conditioned media. Increasing in complexity, the two-chamber system provided by the Transwell insert also lends itself to coculture with an additional cell type in the bottom chamber, such as endothelial or PMC cells (239, 240).

Building upon these simpler systems, advanced models have also been developed that incorporate a three-dimensional culture into a Transwell insert-based platform. These models often include other tissue features such as an ECM/stromal component (189, 243) and immune components (189). For example, Bhowmick et al. created a 3D chitosan-collagen scaffold polymerized on top of a Transwell insert membrane before culturing human small airway epithelial cells on top of this scaffold and airlifting (exposing to air) the model. When compared to a non-3D scaffold control, they found differences in protein expression in uninfected, H1N1, and H3N3 flu virus-infected epithelial cells (243). These results suggest that the addition of ECM components makes a difference in cellular functions.

Additionally, Nguyen Hoang et al. (189) developed a Transwell insert that incorporates both a stromal component and an immune component within the model. In this model, fibroblasts were suspended in type 1 collagen, forming a 3D stromal layer on top of the Transwell insert membrane. Dendritic cells are then cultured on top of this stromal layer prior to epithelial cell culture and airlifting (189). It was found that the capacity of DC to produce chemokines is regulated by the 3D organotypic model, and that soluble components secreted in their lung tissue model induced CCL18 in DC, a function absent in other culture conditions including conditioned media, suggesting that control over the 3D microenvironment is critical for immune system function – an important consideration when designing models for studying host-pathogen interactions. This lung tissue model has been further adapted to incorporate monocytes and macrophages for the study of early granuloma formation in *M. tuberculosis* infection (49, 227, 244). These studies enabled visualization and quantification of immune cell clustering in virulent *M. tuberculosis* infection at the site of infection in the tissue, which is not possible to assess in single-culture systems, thus providing a unique opportunity to study host-pathogen interactions in experimental tissue.

### Limitations of Transwell Insert Models

Transwell insert models are versatile and easily integrated into standard cell culture equipment, supporting their widespread use and adaptation. However, on the spectrum of complexity for *in vitro* models, they are relatively limited in recapitulating the lung tissue microenvironment and function. For example, the membrane separating the compartments is made of a polymeric material and does not fully recapitulate the extracellular matrix environment seen in the lamina propria of lung tissue (226). Therefore, any desired control over 2D or 3D placement of ECM hydrogels or cell types is challenging in Transwell inserts, which are designed for a monolayer or single layer of 3D gel in each compartment. Further, while 96 well Transwell inserts are available, cell insert models tend to require relatively numerous amounts of cells and reagents. This can limit these models to easy-to-obtain cell types, which may not be as biologically relevant as valuable cells, such as primary cells from patients with lung disease. Therefore, meso- or microscale models may be better suited than traditional well-plate culture for applications requiring the use of rare or valuable cell types. Lastly, the diffusion profile of factors between the apical chamber and the basal chamber is poorly characterized, making it difficult to use Transwells for studies where diffusion of signaling factors needs to be tightly controlled (245, 246).

## Microfluidic and Microfabricated *In vitro* Models

Microfluidic and microfabrication techniques have become a useful tool for adding additional control and complexity to *in vitro* models. Microfluidic systems, which typically contain a channel or network of channels with at least one dimension on the sub-millimeter scale, permit more complex control of fluid and airflow, allowing for dynamic culturing conditions (188, 221, 226, 247). Other systems create dynamic culture conditions by adding modular functionalities and components (i.e., different cell types, microorganisms, etc.) at different experimental timepoints (178, 248). Moreover, the ability to shape hydrogels or create microscale compartments offers spatial control of different cell types that have varying levels of communication with each other from diffusion-based signaling to volatile signaling (178, 188, 221, 226). Finally, microfabrication techniques enable microscale culture dimensions and volumes, thus requiring the use of fewer cells than a traditional macroscale (e.g., well-plate) approach (202, 211, 249, 250).

### Dynamic “Breathing” Lung Tissue Models

The microfabricated *in vitro* lung model (the “lung-on-a-chip”) developed by Huh et al. (188) in 2010 has been an influential model over the past decade. A schematic of a “lung-on-a-chip” type model and other models discussed can be found in Table 1. The model features a thin microfabricated porous membrane separating a top and bottom chamber. Alveolar-like epithelial cells are seeded on top of the porous membrane in the top chamber, where an air–liquid interface is created. Endothelial cells are seeded on the basal side of the membrane in the bottom channel where blood or media is flowed. Because it is a closed system, airflow can be controlled both on the apical ALI and flow rate of blood or media can be controlled in the bottom chamber. To add an additional functionality to the model, the design includes two side chambers on either side of the central channels, which can be put under cyclic vacuum or pressure, thus stretching the membrane that contains the cells in the inner channel. By modulating the pressure on the side chambers, the researchers can induce cyclical mechanical strain, similar to the mechanical stretching that alveolar tissue undergoes during breathing (188). This model is versatile and has found widespread use for studying different pathologies such COPD (221), pulmonary edema (251), and cigarette-induced lung damage (222), and has also been adapted to model the small airway where pseudostratified epithelium and mucin producing cells are observed after ALI exposure (221, 222). Notably, the alveolar model has been used as an infection model, where pathogens are introduced to the apical compartment and the effect of this direct contact is measured in the epithelium and endothelium below (188). Conditioned media or other relevant factors can also be introduced into the bottom compartment as a method for investigating soluble factor signaling.

Other models have also been developed with the goal of modeling cyclical mechanical stretching to mimic breathing *in vitro*, such as Stucki et al. (223) who developed a lung model that has actuation functionality. Much like Huh et al. (188) this model cultures alveolar epithelial cells on a thin porous membrane where the bottom side of the membrane is lined with endothelial cells. The actuation is brought by another flexible membrane at the bottom of the media chamber on top of a void channel. When negative pressure is applied in the void channel, the flexible membrane at the bottom of the media channel pulls down, thus pulling down on the flexible membrane containing the epithelial cells. This is similar to how the diaphragm decreases the pressure in the air cavity as breathing occurs *in vivo*. While the concept and materials (i.e., a porous membrane) are similar to the Huh et al. (188) “lung-on-a-chip,” this model differs slightly in geometry of where the actuation occurs, pulling down on the membrane rather than from the side. Further, the upper chamber is open, as it does not have a ceiling, removing the ability to control the apical airflow, but providing pipette access to the culture by users. In fact, human primary pulmonary alveolar cells collected from patients who underwent partial lung resection were utilized in this model, illustrating its applicability for valuable cell populations (223). This model has also been used for applications such as wound healing (224) and has been modified to include microelectronics for impedance measurements (225). Similar to the Huh et al. (188) model, this model can be used for study of cross-talk such as direct or soluble factor signaling, as pathogens can be directly introduced on the apical side or conditioned media can be introduced to the bottom compartment.

### Suspended Microfluidic Small Airway Models

Previously described alveolar models focus on the interaction between epithelial and endothelial cells, as these are the most dominant cell type in that region of the lung. Other small airway microfluidic models, including the “small-airway on a chip” pioneered by Benam et al. (221) in 2016, also looks at the interaction between endothelial and epithelial cells. However, at the level of the small airway, such as the bronchioles, smooth muscle cells are also a relevant component, as the bronchioles will constrict or relax to increase the diameter of the airway, thus regulating airflow to different parts of the lung. Humayun et al. (226) developed a microfabricated small airway model that incorporates smooth muscle cells and epithelial cells. The model looks similar to the small-airway on a chip from Benem et al. (221) in that it contains a top chamber where the epithelial cells are grown, separated by a thin membrane with a bottom chamber that contains a second cell type (in this case smooth muscle cells). However, this model differs in several ways. For one, the cell type in the bottom chamber is smooth muscle cells (SMC) and not endothelial, which enables the researcher to model diseases such as asthma or pulmonary hypertension where SMC are important in disease pathogenesis. Secondly the “membrane” separating the two chambers is a suspended ECM hydrogel, more specifically a mixture of matrigel and collagen. This more closely represents the ECM environment than Transwell inserts or the other *in vitro* models previously discussed that rely on a bioinert polymer such as polydimethylsiloxane (PDMS). Further, this model does not include the mechanical functionality of the other *in vitro* models, as it is not a closed microfluidic system. However, the open microfluidic system offers simple fabrication and pipette accessibility. Since this model represents the functionality of the small airway for bronchoconstriction, it is perhaps better suited for studies of diseases where bronchoconstriction is relevant, such as asthma and COPD. However, it can be used as an interesting model to investigate the cross-talk involvement of smooth muscle cells in infections. Alternatively, this model can be altered such that the smooth muscle cells are replaced with a different cell type (e.g., endothelial cells). Further, because it utilizes a 3D suspended ECM, other cell-types could be encapsulated within the ECM, such as stromal cells or immune components, similar to the inclusion of DC in the Transwell 3D model by Nguyen Hoang et al. (189).

### Microfluidic Lung Tissue Model for Studying Soluble and Volatile Factor Signaling

Apart from models that incorporate mechanical components to stimulate breathing *in vitro*, Barkal et al. (178) developed a platform specifically designed for probing multikingdom cell signaling in the lung microenvironment; this model not only allows for investigating direct and soluble factor cross-talk via placing pathogens in direct contact with the host lung tissue model, but also incorporates functionality for investigating volatile signaling. Moreover, this model incorporates a wide range of cellular components, including a three-dimensional collagen-based ECM with suspended fibroblast cells, where channels for the epithelium and endothelium are cast through, with one larger epithelium channel in the middle and two smaller endothelium channels on either side parallel to the epithelial channel. Similar to the Humayun et al. (226) model, the compartments are not separated by an inert plastic or polymer, such as PDMS, but rather a more biologically relevant hydrogel with the addition of suspended fibroblasts. Further, the air liquid interface is uniquely created in a three-dimensional tubular channel, more similar to *in vivo* bronchial structure (178). The endothelium-lined channels are cast cylindrically in the same manner as the epithelial lined channels. The authors are able to uniquely model a microenvironmental infection by introducing *A. fumigatus* directly into the apical lung epithelial channel and allowing it to grow out into the surrounding ECM. They measure cytokine concentrations in the media in the side endothelial channels in response to this “infection.” The authors further took advantage of the side endothelial channels and ECM by introducing blood immune components to the endothelial channels. Specifically, neutrophils were introduced via capillary flow into the side channels of the *A. fumigatus* infected model and migration into the ECM as a result of infection was measured. Finally, the authors designed an insert that clicks into the lung *in vitro* model chip that can culture two separate fungi or bacteria next to the model, enabling examination of multikingdom volatile signaling by culturing *A. fumigatus* and *P. aeruginosa* in this side insert. Cytokine levels again were measured from the endothelial side channels and shown to differ based on what combination of pathogens were included in the multi-kingdom culture. Notably this model offers multiple functionalities for studying pathogens that can contribute to chronic infections and cross-talk amongst these different cell types and kingdoms within a co-infection scenario. Ultimately, this platform, in addition to those described above, can enable researchers to probe very specific aspects of the lung microenvironment for a wide range of conditions and infections in a controlled and versatile manner.

## Conclusion

Deciphering the complex signaling phenomena of the lung microenvironment, in the context of CPIs, remains a significant challenge due to the myriad of factors contributing to this signaling milieu. The communication amongst host and pathogen cells through secreted factors, physical contact, and even volatile factors, can drive acute and chronic infections as pathogens evade immunity, but it can also control and impede progression of disease through coordinated immune defenses. Further, accounting for the numerous signaling components and mechanisms occurring simultaneously in the lung in chronic infections, the exchange of signals in this microenvironment becomes extremely convoluted, requiring innovative and creative studies to develop an understanding of this space. The recent advances in *in vitro* modeling can help facilitate these studies, as these platforms are continually increasing in complexity and adaptability, enabling researchers to utilize these models in conjunction with *in vivo* studies and to answer specific research queries. Further, the large gap in our understanding surrounding volatile signaling in the lung represents a significant opportunity for fundamental and applied research studies, which can be further facilitated through the use of these novel *in vitro* platforms. Ultimately, we aimed to highlight similar signaling mechanisms in diverse pulmonary infections to elucidate novel connections and similarities between diseases, as well as to paint a picture of the complexity of this signaling environment during infection.

## Author Contributions

SBB and MS conceived of the topic for the manuscript. SBB and AH wrote and organized the manuscript. AT, SB, and MS edited and provided feedback on the manuscript. All authors reviewed and approved the final manuscript.

## Conflict of Interest

The authors declare the following potential conflicts of interest in companies pursuing open microfluidic technologies: AT: Stacks to the Future, LLC. The remaining authors declare that the research was conducted in the absence of any commercial or financial relationships that could be construed as a potential conflict of interest.
